# The *CTGF* gene −945 G/C polymorphism is not associated with cardiac or kidney complications in subjects with type 2 diabetes

**DOI:** 10.1186/1475-2840-11-42

**Published:** 2012-04-26

**Authors:** Sheila K Patel, Bryan Wai, Richard J MacIsaac, Sharon Grant, Elena Velkoska, Michelle Ord, Sianna Panagiotopoulos, George Jerums, Piyush M Srivastava, Louise M Burrell

**Affiliations:** 1 Department of Medicine, Austin Health, University of Melbourne, Level 7, Lance Townsend Building, 145 Studley Road, Melbourne, VIC, 3084, Australia; 2 Department of Cardiology, Austin Health, Melbourne, Australia; 3 Department of Endocrinology and Diabetes, St Vincent's Hospital, Victoria, Australia; 4 Endocrine Centre of Excellence, Austin Health, Melbourne, Australia

**Keywords:** Type 2 diabetes, Connective tissue growth factor, CTGF, Cardiac fibrosis, Chronic kidney disease.

## Abstract

**Background:**

Connective tissue growth factor (CTGF) has been implicated in the cardiac and kidney complications of type 2 diabetes, and the *CTGF −*945 G/C polymorphism is associated with susceptibility to systemic sclerosis, a disease characterised by tissue fibrosis. This study investigated the association of the *CTGF −*945 G/C promoter variant with cardiac complications (left ventricular (LV) hypertrophy (LVH), diastolic and systolic dysfunction) and chronic kidney disease (CKD) in type 2 diabetes.

**Methods:**

The *CTGF −*945 G/C polymorphism (rs6918698) was examined in 495 Caucasian subjects with type 2 diabetes. Cardiac structure and function were assessed by transthoracic echocardiography. Kidney function was assessed using estimated glomerular filtration rate (eGFR) and albuminuria, and CKD defined as the presence of kidney damage (decreased kidney function (eGFR <60 ml/min/1.73 m^2^) or albuminuria).

**Results:**

The mean age ± SD of the cohort was 62 ± 14 years, with a body mass index (BMI) of 31 ± 6 kg/m^2^ and median diabetes duration of 11 years [25^th^, 75^th^ interquartile range; 5, 18]. An abnormal echocardiogram was present in 73% of subjects; of these, 8% had LVH alone, 74% had diastolic dysfunction and 18% had systolic ± diastolic dysfunction. CKD was present in 42% of subjects. There were no significant associations between the *CTGF −*945 G/C polymorphism and echocardiographic parameters of LV mass or cardiac function, or kidney function both before and after adjustment for covariates of age, gender, BMI, blood pressure and hypertension. *CTGF −*945 genotypes were not associated with the cardiac complications of LVH, diastolic or systolic dysfunction, nor with CKD.

**Conclusions:**

In Caucasians with type 2 diabetes, genetic variation in the *CTGF −*945 G/C polymorphism is not associated with cardiac or kidney complications.

## Background

Connective tissue growth factor (CTGF) also known as CCN-2, is a cysteine rich secreted protein that mediates tissue fibrosis in multiple organs including the heart and kidney [1-3]. In the heart, CTGF is expressed in myocytes and fibroblasts [4] and can induce hypertrophy [5], and extracellular matrix production [6]. In man, CTGF gene expression is increased in ischaemic cardiac tissue [4], and both CTGF gene and protein expression are up-regulated in experimental models of cardiac injury and diabetes, and associated with ongoing cardiac fibrosis [5,7-10]. Cardiac fibrosis increases the mechanical stiffness of the heart, which impairs myocardial contractility and contributes to left ventricular (LV) hypertrophy (LVH), and both diastolic and systolic dysfunction [11-13]. Overexpression of CTGF is also thought to play a role in mediating glomerulosclerosis and tubulointerstitial fibrosis, key features of diabetic kidney disease, in both experimental models and in man [14-17].

Cardiac and kidney complications are common in type 2 diabetes, but to date there are limited studies examining the role of CTGF in cardiac or kidney disease in these patients. A number of studies have investigated CTGF in type 1 diabetes [18-20] and shown that CTGF is important in the pathogenesis of kidney disease; plasma CTGF levels correlate with proteinuria and creatinine clearance [18], and can predict end-stage kidney disease and mortality [19], whilst urinary CTGF levels correlate with kidney disease severity [20].

The *CTGF* gene is located on chromosome 6q23.1 and polymorphic sites in the *CTGF* promoter that lie within putative regulatory elements have been identified [21]. Recently, the GG genotype of the *CTGF −*945 G/C promoter polymorphism was shown to be 2.2 fold higher in patients with systemic sclerosis, compared to control subjects, and was associated with increased incidence of lung fibrosis [22]. The C allele at position −945 appears to be critical for the transcriptional suppression of the *CTGF* gene and leads to a reduced CTGF production, whilst substitution with the G allele at −945 results in increased transcription and expression of CTGF [22].

To date, there are limited studies investigating the role of the *CTGF* gene in the complications of type 2 diabetes. We examined the relevance of the *CTFG −*945 G/C promoter polymorphism to the cardiac complications of LVH, diastolic and systolic dysfunction (assessed with echocardiography), and kidney disease assessed with the estimated glomerular filtration rate (eGFR) and 24-hr urinary albumin excretion rate in a cohort of Caucasian subjects with type 2 diabetes.

## Methods

### Research design and methods

#### Study sample

Ethical approval was obtained from the Human Research Ethics Committee at Austin Health, Melbourne and participants provided consent. The study included 495 subjects with type 2 diabetes, prospectively recruited at attendance for transthoracic echocardiography as part of a complications surveillance program at Austin Health, Melbourne, Australia as previously described [13,23]. Subjects of non-European ancestry were excluded.

#### Medical history and clinical measurements

Subjects completed a questionnaire at the time of the echocardiogram, and information on diabetes duration, history of hypertension and ethnic background were obtained. Height and weight were measured for determination of body mass index (BMI) and body surface area. Blood pressure was measured in a semi-recumbent position with a mercury sphygmomanometer with an appropriate size cuff and the average of two measurements was used for analysis. Hypertension was defined as present if participants were on anti-hypertensive medication, had a history of hypertension and/or had evidence of hypertension (clinic blood pressure >130/80 mmHg) [24]. Urinary albumin excretion rate was estimated from a 24-hour urine collection and glycosylated hemoglobin (HbA_1c_) and fasting plasma glucose was measured as previously described [13]. The eGFR was calculated using the four component abbreviated MDRD equation [25]. Microalbuminuria was defined as present if 2 of 3 consecutive urine samples had an albumin excretion rate of ≥20 μg/min but ≤200 μg/min; or macroalbuminuria if 2 out of 3 consecutive urine samples revealed an albumin excretion rate of >200 μg/min. Subjects were considered to have chronic kidney disease (CKD) if they had reduced kidney function (eGFR <60/ml/min/1.73 m^2^) or kidney damage (microalbuminuria or macroalbuminuria) [26,27]. Whole blood was collected for DNA extraction.

#### Echocardiography

Transthoracic echocardiography was performed as previously described using a commercially available ultrasound system (Vivid 9, 3.5 MHz transducer) [13,28,29]. Peak mitral E and A (early and late diastolic peak filling velocities respectively) waves were obtained for calculation of the E/A ratio. Tissue Doppler imaging was used to obtain the peak early diastolic mitral annular velocity (e´) for calculation of the E/e´ ratio. Diastolic dysfunction was classified as present if the subjects had abnormal relaxation, pseudonormal or restrictive physiology patterns [13]. LV systolic function was assessed quantitatively and qualitatively. LV ejection fraction (LVEF) was measured with the biplane Simpson’s method. Systolic dysfunction was defined by a LVEF of <50% and/or the presence of regional wall motion abnormalities. LV mass was indexed to body surface area and LVH was defined as LV mass index >115 g/m^2^ in men and >95 g/m^2^ in women and or posterior wall thickness of >1.2 cm [28].

#### Genotyping

Genomic DNA was extracted using a Nucleon BACC2 DNA kit (GE Healthcare, Australia). The *CTGF −*945 G/C promoter polymorphism (rs6918698) was genotyped using the Sequenom MassARRAY system (Sequenom, San Diego, CA, USA) at the Australian Genome Research Facility (AGRF, Brisbane, Australia). Of the genotyped samples, 10% were duplicates and there were at least four negative controls per 96 well plate. Genotyping accuracy was determined by the genotype concordance between duplicate samples and was 100%.

#### Statistical analyses

All analysis was performed using SPSS version 18 (SPSS Inc., Chicago, IL, USA). The *CTGF −*945 G/C genotype frequency was assessed for Hardy-Weinberg equilibrium using the χ^2^ goodness-of-fit test. Normality was assessed by evaluating quantile-quantile (Q-Q) plots for continuous variables and all Q-Q plots were normal, except for diabetes duration, 24-hr urinary albumin excretion and the E/e´ ratio. Continuous variables are presented as means ± standard deviation (SD) and variables that were not normally distributed are presented as medians and the interquartile range [25^th^ – 75^th^ quartile]. Non-normally distributed variables were log transformed before analyses. The relationship between the *CTGF −*945 G/C polymorphism with continuous variables was examined by univariate general linear model analysis using the additive genetic model. The association of *CTGF −*945 G/C genotypes with continuous variables were examined further using multiple linear regression analysis after adjusting for covariates in the additive genetic model. Covariates affecting echocardiographic and kidney parameters were, age, gender, BMI, systolic blood pressure, diastolic blood pressure and hypertension. The dominant and recessive genetic models were also examined with adjustment for the same covariates. Differences in proportions between those with and without cardiac complications and CKD with the *CTGF −*945 G/C polymorphism was assessed by the Chi-square test. Two-tailed p-values <0.05 were considered significant.

## Results

Table 1 shows the general characteristics, blood pressure, echocardiographic and kidney function variables in the study cohort. The *CTGF −*945 G/C genotype data was available for analysis in 495 subjects (277 men and 218 women) aged 61.9 ± 14.2 years (mean ± SD), with a BMI of 30.7 ± 6.2 kg/m^2^ and median [25^th^, 75^th^ interquartile range] diabetes duration was 11 [5,18] years. Hypertension was present in 82% of the cohort. Echocardiographic data was available for analysis in 445 subjects with the *CTGF −*945 polymorphism. Fifty subjects were excluded due to moderate/severe valve dysfunction, a history of valve replacement/repair or a technically difficult study. An abnormal echocardiogram was found in 73% of the cohort. Of these, 8% had LVH alone, 74% had diastolic dysfunction (with no systolic dysfunction), and 18% had systolic ± diastolic dysfunction. Overall, 49% of subjects had LVH, either alone or with cardiac dysfunction.

**Table 1:** Characteristics of the type 2 diabetes cohort (n = 495).

**Characteristic**	**Subjects with type 2 diabetes**
Age (years)	61.9 ± 14.2
Sex, % male (n)	56% (277)
BMI (kg/m^2^)	30.7 ± 6.2
Diabetes duration (y)^*^	11 [5,18]
Fasting plasma glucose (mmol/l)	9.7 ± 3.8
HbA_1C_ (%)	7.7 ± 1.2
Systolic BP (mmHg)	137 ± 19
Diastolic BP (mmHg)	75 ± 10
Hypertension, (n)	82% (404)
**Abnormal echocardiogram (n)**	73% (325)
LVH alone (n)	8% (27)
Diastolic dysfunction (n)	74% (241)
Systolic ± diastolic dysfunction (n)	18% (57)
**Chronic kidney disease (n)**†	42% (207)
a) eGFR <60 ml/min/1.73 m^2^ (n)	55% (113)
b) eGFR >60 ml/min/1.73 m^2^ (n)	45% (94)
Microalbuminuria (n)	71% (67)
Macroalbuminuria (n)	29% (27)

Data is expressed as mean ± SD, ^*^Median [25th, 75th quartiles] or percentages (numbers). BMI, body mass index; LV, left ventricle. **†**Chronic kidney disease defined as a) eGFR <60 ml/min/1.73 m^2^ or b) presence of albuminuria in those with eGFR >60 ml/min/1.73 m^2^.

CKD was present in 42% of subjects. In the subjects with CKD, 55% had reduced kidney function (eGFR <60 ml/min/1.73 m^2^), and the remainder (eGFR >60 ml/min/1.73 m^2^), had evidence of kidney damage with microalbuminuria in 71% and macroalbuminuria in 29%.

**Figure 1:**
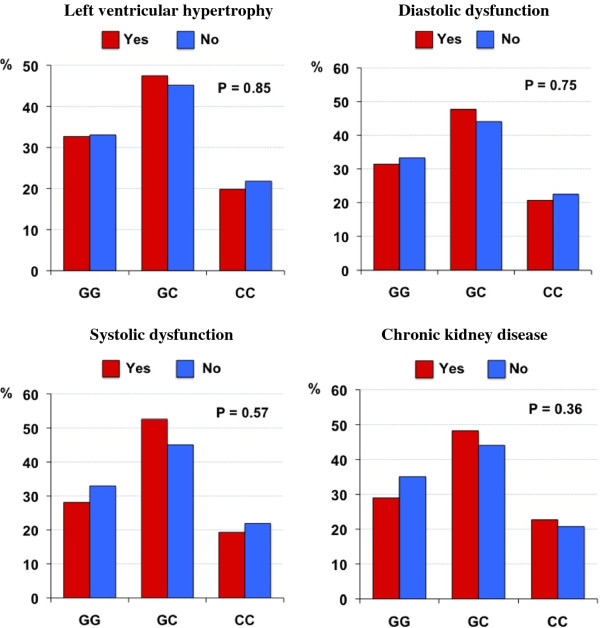
**The presence of cardiac and kidney complications in type 2 diabetes according to the*CTGF −*945 G/C genotype:** figures show the proportion (%) of subjects with and without the complication according to genotype. The P values for differences in proportions are shown.

The frequency of the *CTGF −*945 G and C alleles were 0.56 and 0.44 respectively and were similar to the allele frequency of the CEPH (Utah residents with ancestry from northern and western Europe) studied in the HapMap project [30]. The distributions of genotypes were in Hardy-Weinberg equilibrium (P >0.05). The *CTGF −*945 G/C polymorphism was not associated with echocardiographic parameters of LV mass, nor diastolic (E/A and E/e´ ratio) or systolic function (LVEF) in either univariate or multivariate analyses (Table 2). Adjustment for covariates age, gender, BMI, systolic blood pressure, diastolic blood pressure and hypertension revealed no associations between echocardiographic variables (LV mass, E/A, E/e´, LVEF) with the *CTGF −*945 G/C polymorphism in the additive, dominant or recessive genetic models. The *CTGF −*945 G/C polymorphism was not associated with kidney function parameters of eGFR and 24-hr urinary albumin excretion rate (Table 2) in univariate or multivariate analysis, after adjustment for the same covariates. Although cardiac abnormalities and CKD were common in this cohort of subjects with type 2 diabetes (Table 1), there were no significant differences between the *CTGF −*945 G/C polymorphism and LVH, diastolic and systolic dysfunction or CKD (Figure 1).

**Table 2:** **Relationship between*CTGF −*945 G/C genotypes and cardiac structure, cardiac function and kidney function parameters.**

	**−945 G/C genotypes**			**P values**			
	**GG**	**GC**	**CC**	**Additive model (unadjusted)**	**Additive model (adjusted)†**	**Dominant model (adjusted)†**	**Recessive model (adjusted)†**
n	161	227	107				
***Cardiac structure***							
LV mass index (g/m^2^)	100.2 ± 26.3	98.3 ± 28.2	97.6 ± 23.2	0.73	0.43	0.26	0.90
***Cardiac function***							
Diastolic function, E/A ratio	1.00 ± 0.30	1.00 ± 0.38	1.01 ± 0.38	0.94	0.89	0.84	0.64
E/e´ ratio^*^	10.00 [7.10 - 13.92]	9.89 [7.53 - 19.00]	9.74 [6.8 - 12.98]	0.51	0.55	0.95	0.35
Systolic function, LVEF (%)	67 ± 11	68 ± 12	69 ± 10	0.19	0.32	0.16	0.33
***Kidney function***							
eGFR (ml/min/1.73 m^2^)	76.5 ± 25.9	72.7 ± 25.8	75.4 ± 25.3	0.44	0.19	0.09	0.98
24-hr urinary albumin excretion (μg/min) ^*^	12.4 [8.2 - 44.0]	14.4 [7.8 - 47.8]	12.8 [8.6 - 35.2]	0.74	0.46	0.28	0.92

Data is expressed as mean ± SD, ^*^Median [25th, 75th quartiles]. LVEF, left ventricle ejection fraction; eGFR, estimated glomerular filtration rate. †Genetic models are adjusted for age, gender, BMI, systolic and diastolic blood pressure and hypertension.

## Discussion

This is the first study to investigate the association of the *CTGF −*945 G/C polymorphism with cardiac and kidney complications in subjects with type 2 diabetes. We report no association between the −945 G/C polymorphism and LV mass, diastolic function, systolic function or kidney function, and no association of the *CTGF −*945 genotypes with LVH, diastolic dysfunction, systolic dysfunction or CKD. The cohort had a high prevalence of cardiac abnormalities and CKD, and was both clinically relevant and enriched to study the association of the *CTGF* gene with the parameters under investigation.

A number of animal studies support a role for CTGF in the pathogenesis of cardiac and kidney fibrosis. In a non-diabetic experimental model of cardiomyopathy, the onset of cardiac fibrosis was associated with increased CTGF gene and protein expression, and impaired cardiac function [9]. In experimental type 1 diabetes, CTGF gene and protein expression were increased in the heart, and treatment to reduce fibrosis was associated with down regulation of CTGF [8]. Similarly, CTGF gene expression was increased in the kidney glomeruli of *db/db* mice compared to non-diabetic mice [17]. The increase in CTGF gene expression was observed early in the course of kidney disease, and increased 28-fold with a longer duration of diabetes compared to control mice [17]. In kidney biopsies taken from subjects with diabetes and kidney disease, CTGF gene expression was increased, and correlated with the severity of tubulointerstitial fibrosis [16]. Thus the data from experimental studies suggests that CTGF is a suitable candidate gene for investigation in disease characterised by tissue fibrosis.

Many studies investigating the *CTGF −*945 G/C polymorphism have been in systemic sclerosis, a condition characterized with extensive fibrosis in multiple organs including the heart [31]. The *CTGF −*945 GG genotype was associated with increased susceptibility to systemic sclerosis in a British cohort [22], but this finding has not been replicated in other cohorts with systemic sclerosis of North American, Thai and European ancestry [32-34]. The −945 G allele has been shown to increase transcription activity and expression of CTGF in vitro [22], but to date no genetic study in systemic sclerosis has assessed plasma CTGF levels, or the correlation of plasma levels with the *CTGF −*945 G/C polymorphism.

Most studies to date have been performed in type 1 diabetes. The *CTGF −*945 G/C polymorphism was examined in a small cohort (n = 22) with type 1 diabetes and ‘dead in bed syndrome,’ a cause of sudden death which may have a cardiac cause possibly related to underlying cardiac fibrosis [35]. The study reported no differences in CTGF staining in heart sections in those found with dead in bed syndrome compared to control heart sections, and no difference in the −945 G/C polymorphism genotype frequency compared to 119 healthy control subjects [35].

Plasma CTGF levels are increased in type 1 diabetes, and are associated with end-stage kidney disease [19]. Three studies have assessed the role of the *CTGF* gene in subjects with type 1 diabetes who have kidney disease [36-38]. Two studies were in independent Northern European cohorts, and reported no significant associations with the *CTGF −*945 G/C polymorphism and kidney disease [36,37] as well as other polymorphisms spanning the *CTGF* gene [37]. Dendooven et al. [36] reported that plasma CTGF was increased in type 1 diabetes subjects with kidney disease, compared to those with normoalbuminuria, but there were no significant associations of the −945 G/C polymorphism with plasma CTGF levels or cardiovascular mortality, non-fatal cardiovascular events and end-stage kidney disease. In the third study, significant associations between the G allele of the *CTGF* promoter −20 C/G polymorphism with microalbuminuria were reported in type 1 diabetes [38], but this study did not examine the *CTGF −*945 G/C polymorphism. Other promoter polymorphisms in *CTGF* have been described [21,37], and three of these at promoter positions −650, -484 and −247 are not associated with kidney disease in subjects with type 1 diabetes [37].

There is limited information on *CTGF* gene or plasma levels in type 2 diabetes. A recent report showed the *CTGF −*945 GG genotype was associated with cardiovascular mortality in 99 haemodialysis patients of whom 24% had type 2 diabetes [39]. Although our own data in a larger cohort of subjects does not suggest that the *CTGF −*945 G/C polymorphism influences less severe kidney disease, it will be of interest to follow up this group to assess the effect of the *CTGF −*945 GG genotype on longer-term cardiac and kidney outcomes. A recent European study in over 4000 type 2 diabetes subjects, investigated the rs9493150 *CTGF* polymorphism with the pathogenesis of type 2 diabetes and pancreatic beta cell function [40]. This polymorphism was not associated with an increased risk of diabetes and with beta cell area [40], but the study did not examine other *CTGF* polymorphisms, including the −945 G/C polymorphism investigated in the current study.

The effect of the −945 G/C polymorphism on plasma CTGF levels in type 2 diabetes is currently unknown. One study did measure plasma CTGF levels in 40 subjects with type 2 diabetes in whom cardiac function was also assessed [41], and reported no difference in CTGF levels between those with normal and abnormal diastolic function and no correlation with echocardiographic measures of diastolic function (E/A and E/e´ ratio) and plasma CTGF levels [41].

Early detection of cardiac and kidney complications in diabetes is essential for improving clinical outcomes. The identification of genetic variants associated with cardiac and kidney complications in type 2 diabetes may provide insights into the molecular mechanisms involved and will inform strategies for the prevention and treatment of cardiac and renal complications in diabetes. Recent studies have identified genetic variants that are associated with angiographically defined coronary artery disease (CAD) in subjects with type 2 diabetes [42-44]. The rs1241321 A/G single nucleotide polymorphism in the dimethylarginine dimethylaminohydrolase 1 (*DDAH1)* gene is associated with an increased risk of major adverse cardiac events in 309 Taiwanese subjects with type 2 diabetes and CAD [42]. In Caucasian subjects with CAD, the G allele of the interleukin 18 (*IL18*) gene +183 A/G polymorphism is associated with significantly reduced serum IL-18 levels in subjects with type 2 diabetes (n = 200) compared to subjects without diabetes [43]. A study in 702 white type 2 diabetes subjects reported an association between the tolloid-like 1 (*TLL1*) gene rs1503298l T/C polymorphism and the number of coronary lesions with ≥20% stenosis independently of age, gender and BMI, and the polymorphism was also associated with the extent of CAD in two independent type 2 diabetes cohorts [44]. The significantly associated genetic variants in the *DDAH1IL18* and *TLL1* genes will need to be explored in other ethnic groups, and in larger cohorts of individuals with type 2 diabetes and cardiac disease. Prospective studies are also needed to investigate if the associated genetic variants contribute to adverse cardiac outcomes in type 2 diabetes.

There are several limitations in our study. Firstly, we did not have a direct assessment of cardiac fibrosis. Cardiac magnetic resonance (CMR) is a accurate non-invasive method for assessment of myocardial fibrosis using the late gadolinium enhancement technique [45], but CMR was not available in our cohort, and the gadolinium contrast agent is contraindicated in patients with severe kidney dysfunction. Secondly, we did not measure plasma CTGF levels, which would have been of interest as there is little data on CTGF levels in type 2 diabetes and the −945 G allele has been previously shown to increase transcriptional activity and expression of CTGF [22]. Although a study in type 1 diabetes subjects found plasma CTGF levels to be increased with kidney disease, the *CTGF −*945 G/C polymorphism did not predict CTGF levels [36]. Finally, further studies are needed to investigate the role of *CTGF* gene polymorphisms and associations with CTGF levels in subjects with type 2 diabetes and cardiac disease.

In conclusion, this is the first study to investigate the relationship between the *CTGF −*945 G/C promoter polymorphism and cardiac and kidney disease in a Caucasian population with type 2 diabetes. We report no evidence of an association between the *CTGF −*945 G/C polymorphism and cardiac or kidney disease in subjects with type 2 diabetes.

## Abbreviations

BMI, body mass index; CTGF, connective tissue growth factor; CKD, chronic kidney disease; CMR, Cardiac magnetic resonance; eGFR, estimated glomerular filtration rate; LV, left ventricular; LVH, left ventricular hypertrophy; LVEF, left ventricular ejection fraction; DDAH1, dimethylarginine dimethylaminohydrolase 1 gene; IL18, interleukin 18 gene; TLL1, tolloid-like 1 gene..

## Competing interests

The authors have no conflict of interest to declare.

## Authors’ contributions

S.K.P. designed the study, performed the laboratory work, analysed and interpreted the data, wrote the manuscript, and reviewed and edited the manuscript. B.W. acquired, analysed and interpreted the data, and reviewed and edited the manuscript. R.J.M, S.G., E.V., M.O, S.P. and G.J. acquired the data and reviewed and edited the manuscript. P.M.S. acquired, analysed and reviewed the manuscript. L.M.B. designed the study, interpreted the data, wrote the manuscript and reviewed and edited the manuscript. All authors read and approved the final manuscript.
